# Gray Matter Volume Abnormality in Chronic Pain Patients With Depressive Symptoms: A Systemic Review and Meta-Analysis of Voxel-Based Morphometry Studies

**DOI:** 10.3389/fnins.2022.826759

**Published:** 2022-06-06

**Authors:** Teng Ma, Yuan-Yuan Ji, Lin-Feng Yan, Jia-Ji Lin, Ze-Yang Li, Wen Wang, Jin-Lian Li, Guang-Bin Cui

**Affiliations:** ^1^Functional and Molecular Imaging Key Lab of Shaanxi Province, Department of Radiology, Tangdu Hospital, Fourth Military Medical University, Xi’an, China; ^2^College of Forensic Medicine, Xi’an Jiaotong University, Xi’an, China; ^3^Key Laboratory of Ministry of Public Health for Forensic Science, Xi’an Jiaotong University, Xi’an, China; ^4^Department of Radiology, Chinese PLA General Hospital, Beijing, China

**Keywords:** chronic pain, depressive symptom, voxel-based morphometry, gray matter volume, meta-analysis

## Abstract

**Background:**

Gray matter volume (GMV) alteration in specific brain regions has been widely regarded as one of the most important neuroplasticity features in chronic pain patients with depressive symptoms (CP-D). However, the consistent and significant results were still lacking. Thus, further exploration was suggested to be performed.

**Objectives:**

This study aimed to comprehensively collect the voxel-based morphometry (VBM) studies on GMV alteration between CP-D and healthy controls (HCs). And a systemic review and meta-analysis were made to explore the characteristic brain regions in chronic pain and depression comorbidity.

**Methods:**

Search of PubMed, MEDLINE, Web of Science, and Cochrane Library databases updated to July 13, 2021. The altered GMV between CP-D and HCs in VBM studies was included in this meta-analysis. In total, 18 studies (20 datasets) and 1320 participants (520 patients and 800 HCs) were included. The significant coordinate information (*x*, *y*, *z*) reported in standard space and the effect size (*t-*value or *z*-score) were extracted and analyzed by anisotropic effect size-signed differential mapping (AES-SDM) 5.15 software.

**Results:**

According to the main analysis results, CP-D showed significant and consistent increased GMV in the left hippocampus (HIP. L) and decreased GMV in the medial part of the left superior frontal gyrus (SFG. L, BA 10) compared to HCs. Subgroup analysis showed significant decreased GMV in the medial orbital part of SFG.R (BA 10) in neuropathic pain, as well as significant increased GMV in the right parahippocampal gyrus (PHG.R, BA 35), left hippocampus (HIP.L, BA 20), and right middle frontal gyrus (MFG.R) in musculoskeletal pain. Furthermore, meta-regression showed a positive relationship between the decreased GMV in the medial part of SFG.L and the percentage of female patients.

**Conclusion:**

GMV abnormality in specific brain areas (e.g., HIP.L and SFG) was robust and reproducible, which could be significantly involved in this comorbidity disease. The findings in this study may be a valuable reference for future research.

**Systematic Review Registration:**

[www.crd.york.ac.uk/prospero/].

## Introduction

Chronic pain has been a major problem and a heavy burden on human health for decades (Kuner and Kuner, 2021). It was estimated that about 20∼50% of adults in developed countries suffered from chronic pain (Fayaz et al., 2016; Grace et al., 2021). More than 1 billion people suffered from headaches, low back pain, and neck pain all of which were considered a significant risk of disability (Mills et al., 2019; Ashina, 2020). Many types of chronic pain (e.g., neuropathic pain and fibromyalgia) can induce emotional disorders, and the incidence of which is about 30∼80% (Yalcin and Barrot, 2014). Depression is one of the most common emotional disorders induced by a persistent stress state of chronic pain. The prevalence of this comorbidity in chronic pain patients is 40∼50%(Rizvi et al., 2021). Poor management and treatment outcomes of chronic pain exacerbate depression, which contributes to human suffering and disability (Yalcin and Barrot, 2014; Sheng et al., 2017; Kuner and Kuner, 2021). In turn, bad emotional states worsen pain (Bushnell et al., 2013; Porreca and Navratilova, 2017). Therefore, further studies on the underlying mechanisms and characteristics of neural remodeling are of great significance for the development of clinical diagnosis and treatment.

Voxel-based morphometry (VBM) has been one of the most important methods to explore the brain structure alteration in neuroimaging studies in recent decades (Ashburner and Friston, 2000), which can find the specific abnormality in pathological conditions, including chronic pain and depression. The gray matter volume (GMV) alteration is one of the most important objects of VBM studies (Borsook et al., 2014). Previous studies have shown that the altered GMV in cortical brain areas was associated with both chronic pain and depression (Gustin et al., 2013). However, the results of many neuroimaging studies are inconsistent. For instance, GMV reduction in the amygdala was reported in fibromyalgia patients with anxiety/depressive symptoms (Burgmer et al., 2009). But the GMV of the amygdala in low back pain patients with depressive symptoms was larger than healthy controls (HCs) (Mao et al., 2013). Likewise, increased GMV in the hippocampus has been reported in several studies to play a crucial role in chronic pain (e.g., burning mouth syndrome, fibromyalgia, and muskuloskeletal pain) with depressive symptoms (Khan et al., 2014; Fayed et al., 2017; James et al., 2018). However, some studies have also reported no changes in hippocampal GMV in chronic pain and depression comorbidity (Seminowicz et al., 2013; Mole et al., 2014). Since the findings of GMV alteration in chronic pain patients with depressive symptoms (CP-D) are heterogenous and controversial, a systemic review and meta-analysis are warranted.

In this systemic review and meta-analysis, we aimed to consider the chronic pain-associated depressive disorder and explore the characteristic brain regions involved in the process of chronic pain and depression comorbidity.

## Methods

This study followed the PRISMA statement (Liberati et al., 2009). The detailed checklist is shown in Supplementary Table 1. The protocol of this systemic review and meta-analysis was registered in PROSPERO (CRD42021267592).

### Data Sources, Study Selection, and Quality Assessment

A comprehensive search of studies published from PubMed, MEDLINE, Web of Science, and Cochrane Library databases from its inception to July 13, 2021, was done. Text words: (“chronic pain”) AND (“depressive” OR “depression”) AND (“VBM” OR “voxel-based morphometry” OR “gray matter” OR “gray matter volume”) were used in this study (Mazine et al., 2018). All studies that had been searched were selected by title and abstract in the first step. Residual studies were checked by the detailed information of the article after removing the unrelated studies. In addition, the references of the included studies and relevant review articles were checked for additional relevant studies. The detailed search information is shown in Supplementary Data 1.

Studies that satisfied the following conditions were included in the meta-analysis: (1) Reported the changed GMV in chronic pain patients; all patients were evaluated by the depression-related scale and showed significantly higher depression scores than HCs; (2) compared the GMV between chronic pain patients with HCs; (3) adult participants (age > 18 years old); (4) showed the available coordinates information in standard space [such as Montreal Neurological Institute (MNI) or Talairach (TAL) space] and the effect size (such as *t*-value or *z*-score). Datasets were excluded if they satisfied the following conditions: (1) animal research; (2) children or adolescents; (3) not VBM method; (4) studies did not compare the changed GMV between chronic pain patients and HCs; (5) without depressive symptoms, not chronic pain, or other unrelated studies.

The 12-point checklist involved in the previous meta-analysis of VBM studies was used to assess the quality of each study selected for this meta-analysis (Tang et al., 2020), which is shown in Supplementary Table 2. Literature search, study evaluation, and selection were independently performed by two investigators (TM and Y-YJ), and all controversial discrepancies were resolved by a third investigator (L-FY) for the final decision.

### Voxel-Wise Meta-Analysis for Chronic Pain Patients With Depressive Symptoms

A meta-analysis of GMV differences between CP-D and HCs was conducted with the seed-based d mapping (SDM) software package (version 5.15) in a standard process (Radua et al., 2012, 2014). The SDM approach used effect sizes to combine reported peak coordinates extracted from databases with statistical parametric maps, and it recreated original maps of the effect size of GMV difference between patients and HCs (Chen et al., 2020; Sheng et al., 2020, 2021). The SDM process was briefly described as follows: First, extracted peak coordinates and effect size (e.g., *t*-values and *z*-score) of differences in GMV between CP-D and HCs from each dataset and the *z*-score could be changed to *t*-values with the online tool on SDM website^1^ (Aoki and Inokuchi, 2016; Aoki et al., 2017); a standard MNI map of the GMV differences was then separately recreated for each dataset using an anisotropic Gaussian kernel. The mean map was generated by voxel-wise calculation of the random-effects mean of the dataset maps, weighted by the sample size, intra-dataset variability, and between-dataset heterogeneity. To optimally balance false positives and negatives, we used the default SDM kernel size and thresholds to optimize sensitivity while controlling false positives [full width at half maximum (FWHM) = 20 mm, *P* = 0.005, peak height Z = 1, cluster extent = 10 voxels] (Radua et al., 2012). Then, a quantitative meta-analytic comparison of altered GMV was conducted by calculating differences between patients and controls in each voxel, and the statistical significance was determined using 50 randomization tests (Kolesar et al., 2019; Liu et al., 2021).

Significant peaks located in gray matter (GM) areas were visualized and discussed in detail in this study. Peaks located in white matter (WM) areas were objectively described and discussed in the end. According to the patient’s chronic pain type, we divided them into musculoskeletal pain and neuropathic pain (Fayed et al., 2017; Ashina, 2020; Finnerup et al., 2021). All the above analysis was repeated in subgroups. The visualization of changed GMV was performed with the MRIcroGL software https://www.nitrc.org/plugins/mwiki/index.php/mricrogl:MainPage/home (Chung and Park, 2019, 2020; Kumral et al., 2021; Park and Jung, 2021).

### Analysis of Sensitivity, Heterogeneity, and Publication Bias

Following the mean analysis of the data, a whole-brain voxel-based jackknife sensitivity analysis was performed to test the robustness of the findings by iteratively repeating the same analysis, excluding one dataset each time. This analysis was to establish the extent to which the results could be replicated. If a brain region remained significant in all or most of (>50%) the combinations of studies, the finding would be considered highly replicable. Heterogeneity analysis was conducted using a random-effects model with Q statistics to explore unexplained between-study variability in the results. Heterogeneous brain regions were obtained using the default SDM kernel size and thresholds. In addition, publication bias was assessed with Egger’s test of the AES-SDM default by extracting the values from statistically significant relevant peaks between patients and HCs. The visualization of heterogeneity information was performed with GraphPad Prism software (version 9). The publication bias was visualized with a funnel plot performed by SDM default in Figure 4.

### Meta-Regression Analyses

Meta-regression was made by the linear model (Select linear model-Meta-regression) and choose the threshold (Probability = 0.0005; Peak height threshold = 1.000; Extent threshold = 10), the correlation between SDM-estimate and factors (age, disease duration, female percentage of patients, and depression score) (Picó-Pérez et al., 2020; Tang et al., 2020).

## Results

### General Information of the Included Studies

**FIGURE 1:**
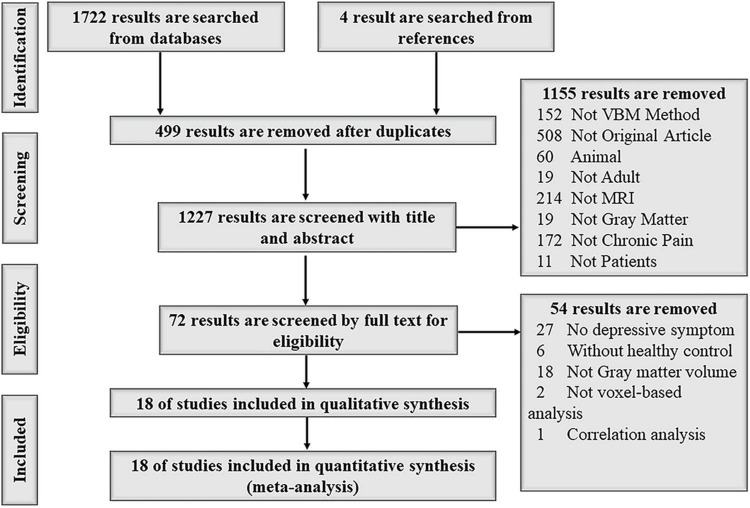
The systemic search followed the PRISMA standard flow diagram.

A total of 18 studies, including three fibromyalgia (Burgmer et al., 2009; Robinson et al., 2011; Fayed et al., 2017), two mixed pain (Seminowicz et al., 2013; Ikeda et al., 2018), three back pain (Schmidt-Wilcke et al., 2006; Mao et al., 2013; Fritz et al., 2016), two trigeminal neuralgia (Wang et al., 2017; Zhang et al., 2018), one knee osteoarthritis (Liao et al., 2018), one musculoskeletal pain (James et al., 2018), one pelvic pain (As-Sanie et al., 2012), one burning mouth syndrome (Khan et al., 2014), one chronic facial pain (Schmidt-Wilcke et al., 2010), one neuropathic pain (Mole et al., 2014), and two migraines (Hubbard et al., 2014; Neeb et al., 2017), and a total of 1,320 participants (520 patients and 800 HCs) were included after systemic searching and selecting from 1,726 studies. The detailed flow diagram is shown in Figure 1. In subgroups, 4 of 18 studies were classified as musculoskeletal pain (Burgmer et al., 2009; Robinson et al., 2011; Fayed et al., 2017; James et al., 2018) and 10 of 18 studies were classified as neuropathic pain (Schmidt-Wilcke et al., 2006, 2010; Mao et al., 2013; Hubbard et al., 2014; Khan et al., 2014; Mole et al., 2014; Fritz et al., 2016; Neeb et al., 2017; Wang et al., 2017; Zhang et al., 2018). General clinical information (authors, publication date, participants, chronic pain types, depression rating, magnetic field, standard space, *P*-value, disease duration, and age) of the included studies was fully collected in Table 1. The detailed depression information of studies included is shown in Supplementary Table 3.

**TABLE 1:** Demographic information of the total 18 studies included.

References	Subjects (F)			Types of chronic pain	Depression (PT, Mean)		Magnetic field (T)	Standard space	*P*-value	Disease duration (Mean, Month)	Age (Mean ± sd.)	
	Total	PT	HC		Scales	Score					PT	HC
David et al., 2013	26(20)	13(10)	13(10)	Mixed chronic pain	BDI	19.85	3.0	MNI	0.05(FWE)	NA	51.4(11.8)	51.6(11.9)
Fayed et al., 2017	23(15)	12(8)	11(7)	Fibromyalgia	HADS	5.90	1.5	MNI	0.05(FWE)	NA	41.7(7.3)	43.3(5.1)
Ikeda et al., 2018	40(25)	23(15)	17(10)	Mixed chronic pain	BDI	16.50	3.0	MNI	0.001(uncorrected)	103	47.7(14.1)	42.3(11.9)
James et al., 2018	105(78)	74(60)	31(18)	Musculoskeletal pain	BDI	15.36	3.0	MNI	0.05(FWE)	132	45.7(13.03)	44.12(13.15)
Liao et al., 2018	60(52)	30(26)	30(26)	Knee osteoarthritis pain	HAMD	6.0*	3.0	MNI	0.05(FWE)	87.6	56.5(6.8)	55.2(5.7)
Mao et al., 2013	60(40)	30(20)	30(20)	Low back pain	HAMD	8.00	3.0	MNI	0.05(FWE)	93.6	51.6(8.6)	50.2(5.8)
	30(20)	15(10)	15(10)	Upper back pain		9.60					49.2(10.1)	49.2(5.6)
Markus et al., 2009	28(28)	14(14)	14(14)	Fibromyalgia	HADS	16.90	3.0	MNI	0.001(uncorrected)	NA	51.0(7.3)	46.9(6.8)
Michael et al., 2011	25(25)	14(14)	11(14)	Fibromyalgia	BDI	34.38	3.0	TAL	0.02(FDR)	NA	43.1(6.9)	42.4(9.8)
Mole et al., 2014	36(NA)	18(NA)	18(NA)	Neuropathic pain	BDI	12.39	3.0	MNI	0.001(uncorrected)	133.2	51.3(NA)	NA
Sawsan et al., 2012	34(34)	17(17)	17(17)	Chronic pelvic pain with endometriosis	CES-D	12.50	3.0	MNI	0.001(uncorrected)	NA	26.1(1.5)	25.9(1.6)
	18(18)	6(6)	12(12)	Chronic pelvic pain without endometriosis		8.80				NA	24.2(1.9)	24.8(1.2)
Shariq et al., 2014	18(18)	9(9)	9(9)	Burning mouth syndrome	BDI	9.10	3.0	MNI	0.05(corrected)	48	54(7.7)	56(8.2)
Tobias et al., 2010	22(18)	11(9)	11(9)	Chronic facial pain	BDI	11.38	1.5	NO	0.05(corrected)	58.27	52.2(8.9)	51.3(8.6)
Wang et al., 2017	76(44)	38(22)	38(22)	Trigeminal neuralgia	HAMD	4.24	3.0	MNI	0.05(FDR)	84.6	55.87(8.38)	55.89(8.06)
Tobias et al., 2006	36(NA)	18(9)	18(NA)	Chronic back pain	HAMD	10.00	1.5	TAL	0.001(uncorrected)	176	50.4(6.8)	49.9(8.7)
Zhang et al., 2018	63(40)	29(19)	34(21)	Trigeminal neuralgia	HAMD	3.79	3.0	MNI	0.05(FWE)	72.24	48.14(11.89)	43.3(10.1)
Fritz et al., 2016	543(263)	111(78)	432(185)	Chronic back pain	PHQ9	4.49	1.5	MNI	0.05(FWE)	NA	53.12(11.77)	48.92(13.96)
Hubbard et al., 2014	35(27)	17(13)	18(14)	Chronic Migraine	POMS	17.35	3.0	MNI	0.05(corrected)	NA	41.71(12.20)	38.89(11.25)
Neeb et al., 2017	42(30)	21(15)	21(15)	Chronic Migraine	BDI	13.00	3.0	MNI	0.05 (FWE)/0.001 (uncorrected)	293.16	49.04(7.46)	49.40(7.79)

BDI, Beck Depression Inventory; HADS, Hospital Anxiety Depression Scale; HAMD, Hamilton Depression Rating Scale; CES-D, Center for Epidemiologic Studies Depression Scale; PHQ9, Patient Health Questionnaire; POMS, Profile of Mood States; NO, Other standard coordinate space; NA, unavailable; PT, patient; HC, healthy control; F, Female; FWE, Family Wise Error; FDR, False Discovery Rate; * = Median.

### Gray Matter Volume Abnormality of Main Analysis in a Total of 18 Studies

**FIGURE 2:**
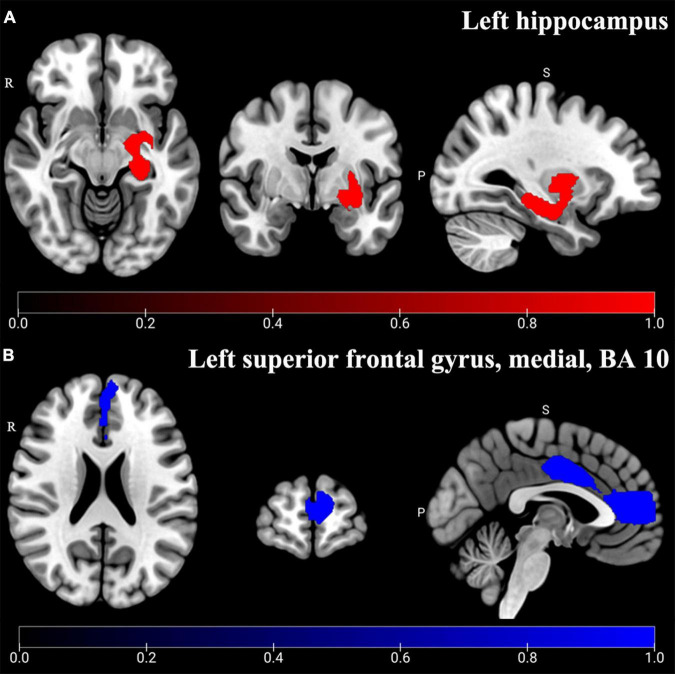
Altered GMV of main analysis results. Significant and consistent increased GMV (Red) in the left hippocampus **(A)** and decreased GMV (Blue) in the left superior frontal gyrus, medial, and BA 10 **(B)** were shown in CP-D compared with HCs (*P* < 0.005).

**FIGURE 3:**
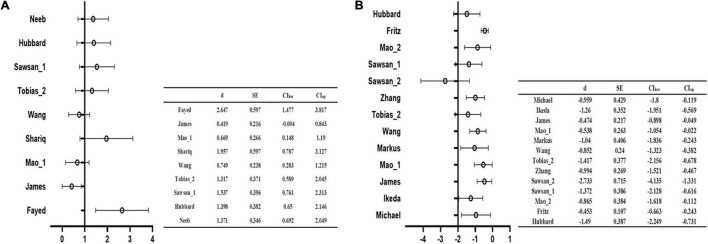
The forest plot of the main analysis results in heterogeneity assessment. Q test of AES-SDM default showed the main results existed heterogeneity in both positive **(A)** and negative **(B)** coordinate results (*P* < 0.05).

**FIGURE 4:**
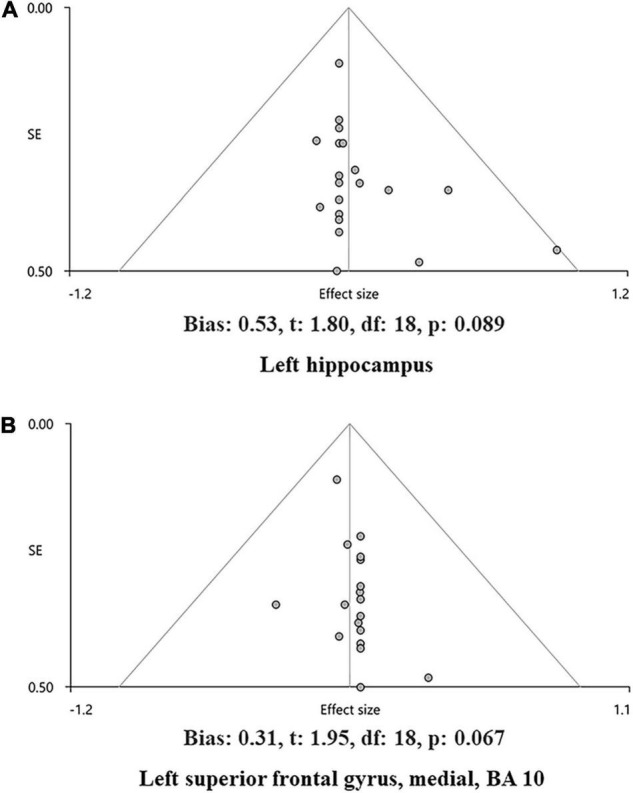
The funnel plot of the main analysis results in bias assessment. Main analysis results in the left hippocampus **(A)** and the left superior frontal gyrus, medial, and BA 10 **(B)** and showed no bias by Egger’s test of AES-SDM default (*P* > 0.05).

The main analysis of 18 studies included showed the altered GMV of two major brain areas could be significant and consistent (Table 2). The increased GMV in the left hippocampus (HIP.L) (MNI: *x* = –24, *y* = –18, *z* = –12, SDM-Z = 1.283, *P* < 0.005) and decreased GMV in the medial part of the left superior frontal gyrus (SFG.L) (BA 10, MNI: *x* = –4, *y* = 62, *z* = 10, SDM-Z = –2.436, *P* < 0.005). Information on brain areas is shown in Figures 2A,B. The heterogeneity analysis of the 18 studies showed heterogeneity between studies (Positive peaks: Q = 24.798, *P* < 0.005; Negative peaks: Q = 30.891, *P* < 0.005) (Supplementary Table 4 and Figures 3A,B). The sensitivity analysis of the main results showed 16 of 20 times of analyses were significant in the HIP.L and 19 of 20 times of analyses were significant in the medial part of SFG.L (Supplementary Table 5). Egger’s test for main results showed no publication bias in HIP.L and the medial part of SFG.L (BA 10) (*P* > 0.05) (Figures 4A,B).

**TABLE 2:** Significant and consistent coordinate results of the main analysis.

MNI coordinate	SDM-Z	P	Voxels	Description	Sub-peak areas	Jackknife analysis
**Positive**						
–24, –18, –12	1.283	< 0.005	1060	Left hippocampus	Left hippocampus Left striatum Left pons Left striatum	16/20
**Negative**						
–4, 62, 10	–2.436	< 0.005	2117	Left superior frontal gyrus, medial, BA 10	Left superior frontal gyrus, medial, BA 10 Right superior frontal gyrus, medial orbital, BA 10 Corpus callosum ^Δ^ Right striatum Left median cingulate/paracingulate gyri, BA 23 Left anterior cingulate/paracingulate gyri, BA 32	19/20
–28, 28, –4	–2.246	< 0.005	80	Left inferior network, uncinate fasciculus ^Δ^	Left inferior network, uncinate fasciculus ^Δ^	19/20

*GMV abnormality in the total 18 studies included (CP-D vs. HCs). **Δ:** White matter area, BA, Brodmann area.*

### Gray Matter Volume Abnormality of Different Types of Pain by Subgroup Analysis

**FIGURE 5:**
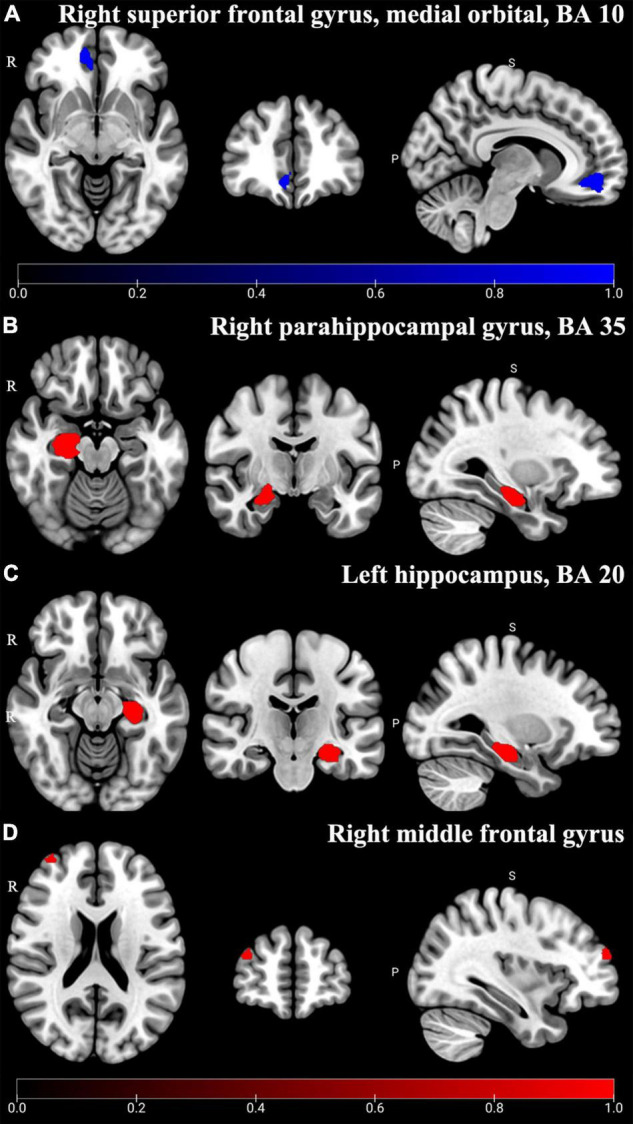
Altered GMV of subgroup analysis results. In CNP-D, the significant decreased (Blue) GMV was shown in the right superior frontal gyrus, medial orbital, and BA 10 **(A)** (*P* < 0.005); in CMP-D, the significant increased (Red) GMV was shown in the right parahippocampal gyrus, BA 35 **(B)**, left hippocampus, BA 20 **(C)**, and right middle frontal gyrus **(D)** (*P* < 0.005).

Chronic neuropathic pain patients with depressive symptoms (CNP-D) showed significant decreased GMV in the medial orbital part of SFG.R compared to HCs (BA 10, MNI: *x* = 12, *y* = 48, *z* = –8, SDM-Z = –2.685, *P* < 0.005) (Table 3 and Figure 5A). Jackknife analysis showed that 8 of 11 times were significant in the medial orbital part of SFG.R, which is shown in Supplementary Table 6.

**TABLE 3:** Significant and consistent results of subgroup analysis.

(A) GMV abnormality in neuropathic pain (CNP-D vs. HCs)					

MNI coordinate	SDM-Z	P	Voxels	Description	Jackknife analysis
**Positive**					
34, -10, -10	1.458	< 0.005	938	Right inferior network, inferior fronto-occipital fasciculus^Δ^	8/11
**Negative**					
12, 48, -8	–2.685	< 0.005	254	Right superior frontal gyrus, medial orbital, BA 10	8/11
-28, 28, -4	–2.697	< 0.005	93	Left inferior network, uncinate fasciculus ^Δ^	10/11

**(B) GMV abnormality in musculoskeletal pain (CMP-D vs. HCs)**					

**MNI coordinate**	**SDM-Z**	**P**	**Voxels**	**Description**	**Jackknife analysis**

**Positive**					
22, -18, -18	1.184	< 0.005	619	Right parahippocampal gyrus, BA 35	3/4
-28, -20, -16	1.155	< 0.005	343	Left hippocampus, BA 20	3/4
36, 58, 22	1.347	< 0.005	46	Right middle frontal gyrus	3/4
**Negative**					
34, 20, 28	–1.500	< 0.005	99	Right superior longitudinal fasciculus II ^Δ^	3/4

***Δ:** White matter area, BA, Brodmann area.*

In addition, chronic musculoskeletal pain patients with depressive symptoms (CMP-D) showed significant increased GMV in the right parahippocampal gyrus (PHG.R) (BA 35, MNI: *x* = 22, *y* = –18, *z* = –18, SDM-Z = 1.184, *P* < 0.005), HIP.L (BA 20, MNI: *x* = –28, *y* = –20, *z* = –16, SDM-Z = 1.155, *P* < 0.005), and right middle frontal gyrus (MFG.R) (MNI: *x* = 36, *y* = 58, *z* = 22; SDM-Z = 1.347, *P* < 0.005) compared to HCs (Table 3 and Figures 5B–D). Jackknife analysis showed that 3 of 4 times were significant in the PHG.R, 3 of 4 times were significant in the HIP.L, and 3 of 4 times were significant in the MFG.R, which are shown in Supplementary Table 6.

### Meta-Regression Analysis

**FIGURE 6:**
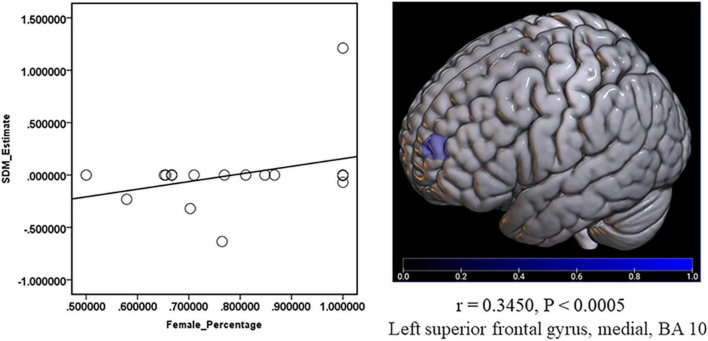
Meta-regression about the relationship between GMV and the percentage of female patients. The percentage of female patients showed a positive relationship with the decreased (Blue) GMV in the left superior frontal gyrus, medial, and BA 10 (*r* = 0.3450, *P* < 0.0005).

The risk factors, including age, sex ratio (female), BDI score, HAMD score, and disease duration, were checked with meta-regression. And the result showed a positive relationship between the decreased GMV in the medial part of SFG.L and the percentage of female patients (*r* = 0.3450, *P* < 0.0005) (Figure 6). No significant results were shown in the other risk factors.

## Discussion

In this study, depressive symptoms accompanying chronic pain were taken into consideration to explore the characteristic brain regions in this comorbid state. By extracting the significant peak coordinates of GMV alteration in different types of chronic pain and checking the depression scales, we found that the increased GMV in the HIP.L and decreased GMV in the medial part of SFG.L were significant and consistent in CP-D. In addition, the sex ratio (female) of patients was positively related to the decreased GMV in the medial part of SFG.L in meta-regression, which indicated that this factor might affect the brain structure in the process of CP-D.

Chronic pain and depression are generally divided into separate diseases in clinical experience (Rotenstein et al., 2016; Park and Zarate, 2019; Grace et al., 2021). However, accumulating studies found that similar brain areas were involved in the common mechanism of both diseases (Han and Pae, 2015; Sheng et al., 2017; Georgopoulos et al., 2019). In the main analysis of our study, CP-D showed a significant decreased GMV in the medial part of SFG.L compared to HCs. And the subgroup analysis also found that the decreased GMV in the medial orbital part of SFG.R was involved in the CNP-D, which was consistent with the results in previous systemic reviews and meta-analyses of neuropathic pain (Pan et al., 2015). Similarly, structure magnetic resonance imaging (sMRI) studies reported that the patients with depression had a significant GMV reduction in the frontal and orbitofrontal cortex (Kraus et al., 2017), as well as a decreased GMV in the medial orbital gyrus (Kandilarova et al., 2019). Thus, we could speculate that the specific brain region of SFG (medial) may play a crucial role in the process of chronic pain and depression comorbidity.

In addition to the congruent abnormality of specific brain areas in both chronic pain and depression, inconsistent brain regions remain (e.g., hippocampus, PHG.R, and MFG.R). In 2017, increased GMV in the bilateral hippocampus was found in fibromyalgia patients compared to HCs (Fayed et al., 2017), which was also shown in patients with burning mouth syndrome (Khan et al., 2014). Besides, previous systemic reviews and meta-analyses demonstrated the increased GMV in the HIP. R was involved in the process of chronic pain (Smallwood et al., 2013). Interestingly, neuroimaging studies reported that depressed patients have smaller hippocampal volumes than HCs (Kraus et al., 2017; Serrano-Sosa et al., 2020). However, the main and subgroup analysis in our study found increased GMV in the HIP. L was significantly involved in CP-D. Similarly, subgroup analysis showed that the significant increased GMV in PHG.R and MFG.R was involved in CMP-D. But previous systemic review and meta-analysis studies reported that patients with fibromyalgia had smaller GMV in PHG.R than HCs (Shi et al., 2016). The previous study has also reported decreased GMV in the MFG. R of patients with MDD, whereas increased GMV in the MFG and R of patients with bipolar disorder (BD) (Wise et al., 2017). Taken together, the GMV alteration in the hippocampus, PHG.R, and MFG.R may be heterogenous and controversial in CP-D. Therefore, it is essential to comprehensively explore the neuromechanisms of these brain regions in CP-D in future studies.

Furthermore, the main and subgroup analysis also revealed that some peak coordinates outside the gray matter mask appeared in WM areas, including the left inferior network, uncinate fasciculus (UF. L), right inferior network, inferior fronto-occipital (IFOF. R), and right superior longitudinal fasciculus II (SLF II. R). On one hand, calculations of voxels were not restricted within the specific masks by the algorithm of SDM, which might cause the peaks to be outside the mask in the recreated map (Radua et al., 2014); on the other hand, the potential function of the significant peaks in WM areas should not be ignored just because it might be due to an error. For instance, emerging evidence reported that the neurobiological significance of low-frequency BOLD fluctuations (LFBFs) was not just shown in GM but also in WM (Ji et al., 2017), and which has been explored in the Parkinson’s disease (PD) (Ji et al., 2018, 2021). Therefore, these findings extending to the WM areas can be accepted cautiously, and can also be learned in conjunction with the adjacent GM areas involved in CP-D. In addition, meta-regression indicated that the sexual difference might affect the GMV in the medial part of SFG.L in CP-D. However, other risk factors should not be ignored because of the limited data, which was suggested to be explored in future.

There were some limitations in this study. First, only four databases were searched. And the studies included were published, while unpublished studies and potential bias could not be ignored; Second, only the peak coordinate information reported in the studies was extracted to be analyzed, which was the common shortage in neuroimaging meta-analysis (Liu et al., 2021); Third, lack of data on the risk factors in meta-regression; Fourth, we only focused on the GMV abnormality to reduce bias. However, further studies should take a broader approach to studying this comorbid disease.

## Conclusion

In conclusion, this systemic review and meta-analysis provide some specific brain areas involved in chronic pain and depressive symptoms comorbidity. The GMV alteration in SFG is consistent with previous studies, which show a stable reduction in CP-D. Although some results of this study are inconsistent with previous studies (especially the hippocampus), the important value of which CP-D cannot be ignored. Furthermore, it is recommended to further explore the neural mechanisms of the hippocampus in CP-D in the future.

## Data Availability Statement

The original contributions presented in the study are included in the article/Supplementary Material, further inquiries can be directed to the corresponding authors.

## Author Contributions

G-BC, J-LL, and WW made the study and article framework design. G-BC, J-LL, and L-FY provided the funding support. TM, Y-YJ, and L-FY performed the systemic search, study selection, data analysis, and wrote the original draft. Z-YL checked the methods and results and provided the technical support of software. G-BC, J-LL, J-JL, and WW revised the article. All authors contributed to the article and approved the submitted version.

## Conflict of Interest

The authors declare that the research was conducted in the absence of any commercial or financial relationships that could be construed as a potential conflict of interest.

## Publisher’s Note

All claims expressed in this article are solely those of the authors and do not necessarily represent those of their affiliated organizations, or those of the publisher, the editors and the reviewers. Any product that may be evaluated in this article, or claim that may be made by its manufacturer, is not guaranteed or endorsed by the publisher.

## Footnotes

1
www.sdmproject.com

## References

[B1] Aoki Y. Cortese S. Castellanos F. X. (2017). Research Review: diffusion tensor imaging studies of attention-deficit/hyperactivity disorder: meta-analyses and reflections on head motion. *J. Child Psychol. Psychiatry* 59 193–202. 10.1111/jcpp.12778 28671333

[B2] Aoki Y. Inokuchi R. (2016). A voxel-based meta-analysis of diffusion tensor imaging in mild traumatic brain injury. *Neurosci. Biobehav. Rev.* 66 119–126. 10.1016/j.neubiorev.2016.04.021 27133211

[B3] Ashburner J. Friston K. J. (2000). Voxel-Based morphometry—the methods. *NeuroImage* 11 805–821. 10.1006/nimg.2000.0582 10860804

[B4] Ashina M. (2020). Migraine. *N. Engl. J. Med.* 383 1866–1876. 33211930 10.1056/NEJMra1915327

[B5] As-Sanie S. Harris R. E. Napadow V. Kim J. Neshewat G. Kairys A. (2012). Changes in regional gray matter volume in women with chronic pelvic pain: a voxel-based morphometry study. *Pain* 153 1006–1014. 10.1016/j.pain.2012.01.032 22387096 PMC3613137

[B6] Borsook D. Erpelding N. Becerra L. (2014). Losses and gains: chronic pain and altered brain morphology. *Expert Rev. Neurother.* 13 1221–1234. 10.1586/14737175.2013.846218 24164053

[B7] Burgmer M. Gaubitz M. Konrad C. Wrenger M. Hilgart S. Heuft G. (2009). Decreased gray matter volumes in the Cingulo-Frontal cortex and the amygdala in patients with fibromyalgia. *Psychosom. Med.* 71 566–573. 10.1097/PSY.0b013e3181a32da0 19414621

[B8] Bushnell M. C. Ceko M. Low L. A. (2013). Cognitive and emotional control of pain and its disruption in chronic pain. *Nat. Rev. Neurosci.* 14 502–511. 10.1038/nrn3516 23719569 PMC4465351

[B9] Chen E. Y. Eickhoff S. B. Giovannetti T. Smith D. V. (2020). Obesity is associated with reduced orbitofrontal cortex volume: a coordinate-based meta-analysis. *Neuroimage Clin.* 28:102420. 10.1016/j.nicl.2020.102420 32961404 PMC7509458

[B10] Chung B. S. Park J. S. (2019). Real-Color volume models made from Real-Color sectioned images of visible korean. *J. Korean Med. Sci.* 34:e86. 10.3346/jkms.2019.34.e86 30886552 PMC6417999

[B11] Chung B. S. Park J. S. (2020). Automatic segmentation of true color sectioned images using FMRIB Software Library: first trial in brain, gray matter, and white matter. *Clin. Anat.* 33 1197–1203. 10.1002/ca.23564 31943396

[B12] Fayaz A. Croft P. Langford R. M. Donaldson L. J. Jones G. T. (2016). Prevalence of chronic pain in the UK: a systematic review and meta-analysis of population studies. *BMJ Open* 6:e10364. 10.1136/bmjopen-2015-010364 27324708 PMC4932255

[B13] Fayed N. Garcia-Marti G. Sanz-Requena R. Marti-Bonmati L. Garcia-Campayo J. (2017). Difference in regional brain volume between fibromyalgia patients and Long-Term meditators. *Actas Esp. Psiquiatr.* 45 268–276. 29199761

[B14] Finnerup N. B. Kuner R. Jensen T. S. (2021). Neuropathic pain: from mechanisms to treatment. *Physiol. Rev.* 101 259–301. 10.1152/physrev.00045.2019 32584191

[B15] Fritz H. McAuley J. H. Wittfeld K. Hegenscheid K. Schmidt C. O. Langner S. (2016). Chronic back pain is associated with decreased prefrontal and anterior insular gray matter: results from a Population-Based cohort study. *J. Pain* 17 111–118. 10.1016/j.jpain.2015.10.003 26476265

[B16] Grace P. M. Tawfik V. L. Svensson C. I. Burton M. D. Loggia M. L. Hutchinson M. R. (2021). The neuroimmunology of chronic pain: from rodents to humans. *J. Neurosci.* 41 855–865. 10.1523/JNEUROSCI.1650-20.2020 33239404 PMC7880288

[B17] Gustin S. M. Peck C. C. Macey P. M. Murray G. M. Henderson L. A. (2013). Unraveling the Effects of Plasticity and Pain on Personality. *J. Pain* 14 1642–1652. 10.1016/j.jpain.2013.08.005 24290444

[B18] Han C. Pae C. (2015). Pain and depression: a neurobiological perspective of their relationship. *Psychiatry Investig.* 12:1. 10.4306/pi.2015.12.1.1 25670939 PMC4310906

[B19] Hubbard C. S. Khan S. A. Keaser M. L. Mathur V. A. Goyal M. Seminowicz D. A. (2014). Altered brain structure and function correlate with disease severity and pain catastrophizing in migraine patients. *ENeuro* 1 e14–e20. 10.1523/ENEURO.0006-14.2014 25893216 PMC4399775

[B20] Ikeda E. Li T. Kobinata H. Zhang S. Kurata J. (2018). Anterior insular volume decrease is associated with dysfunction of the reward system in patients with chronic pain. *Eur. J. Pain* 22 1170–1179. 10.1002/ejp.1205 29436061

[B21] James H. B. Marina S. A. Antoni K. A. Sarah C. A. Richard W. C. D. (2018). Structural network differences in chronic muskuloskeletal pain: beyond fractional anisotropy. *NeuroImage* 182 441–455. 10.1016/j.neuroimage.2017.12.021 29242104

[B22] Ji G. Liao W. Chen F. Zhang L. Wang K. (2017). Low-frequency blood oxygen level-dependent fluctuations in the brain white matter: more than just noise. *Sci. Bull.* 62 656–657. 10.1016/j.scib.2017.03.021 36659309

[B23] Ji G. J. Liu T. Li Y. Liu P. Sun J. Chen X. (2021). Structural correlates underlying accelerated magnetic stimulation in Parkinson’s disease. *Hum. Brain Mapp.* 42 1670–1681. 10.1002/hbm.25319 33314545 PMC7978118

[B24] Ji G. J. Ren C. Li Y. Sun J. Liu T. Gao Y. (2018). Regional and network properties of white matter function in Parkinson’s disease *Hum. Brain Mapp.* 40 1253–1263. 10.1002/hbm.24444 30414340 PMC6865582

[B25] Kandilarova S. Stoyanov D. Sirakov N. Maes M. Specht K. (2019). Reduced grey matter volume in frontal and temporal areas in depression: contributions from voxel-based morphometry study. *Acta Neuropsychiatr.* 31 252–257. 10.1017/neu.2019.20 31234950

[B26] Khan S. A. Keaser M. L. Meiller T. F. Seminowicz D. A. (2014). Altered structure and function in the hippocampus and medial prefrontal cortex in patients with burning mouth syndrome. *Pain* 155 1472–1480. 10.1016/j.pain.2014.04.022 24769366

[B27] Kolesar T. A. Bilevicius E. Wilson A. D. Kornelsen J. (2019). Systematic review and meta-analyses of neural structural and functional differences in generalized anxiety disorder and healthy controls using magnetic resonance imaging. *Neuroimage Clin.* 24:102016. 10.1016/j.nicl.2019.102016 31835287 PMC6879983

[B28] Kraus C. Castren E. Kasper S. Lanzenberger R. (2017). Serotonin and neuroplasticity - Links between molecular, functional and structural pathophysiology in depression. *Neurosci. Biobehav. Rev.* 77 317–326. 10.1016/j.neubiorev.2017.03.007 28342763

[B29] Kumral E. Bayam F. E. Özdemir H. N. (2021). Cognitive and behavioral disorders in patients with precuneal infarcts. *Eur. Neurol.* 84 157–167. 10.1159/000513098 33827093

[B30] Kuner R. Kuner T. (2021). Cellular circuits in the brain and their modulation in acute and chronic pain. *Physiol. Rev.* 101 213–258. 10.1152/physrev.00040.2019 32525759

[B31] Liao X. Mao C. Wang Y. Zhang Q. Cao D. Seminowicz D. (2018). Brain gray matter alterations in Chinese patients with chronic knee osteoarthritis pain based on voxel-based morphometry. *Medicine* 97:e145. 10.1097/MD.0000000000010145 29561420 PMC5895331

[B32] Liberati A. Altman D. G. Tetzlaff J. Mulrow C. Gøtzsche P. C. Ioannidis J. P. (2009). The PRISMA statement for reporting systematic reviews and meta-analyses of studies that evaluate healthcare interventions: explanation and elaboration. *BMJ* 339:b2700. b2700 10.1136/bmj 19622552 PMC2714672

[B33] Liu X. Lai H. Li J. Becker B. Zhao Y. Wang S. (2021). Gray matter structures associated with neuroticism: a meta-analysis of whole-brain voxel-based morphometry studies. *Hum. Brain Mapp.* 42 2706–2721. 10.1002/hbm.25395 33704850 PMC8127153

[B34] Mao C. Wei L. Zhang Q. Liao X. Yang X. Zhang M. (2013). Differences in brain structure in patients with distinct sites of chronic pain: a voxel-based morphometric analysis. *Neural Regen. Res.* 8 2981–2990. 10.3969/j.issn.1673-5374.2013.32.001 25206618 PMC4146206

[B35] Mazine A. Rocha R. V. El-Hamamsy I. Ouzounian M. Yanagawa B. Bhatt D. L. (2018). Ross Procedure vs Mechanical Aortic Valve Replacement in Adults: a Systematic Review and Meta-analysis. *JAMA Cardiol.* 3:978. 10.1001/jamacardio.2018.2946 30326489 PMC6233830

[B36] Mills S. E. E. Nicolson K. P. Smith B. H. (2019). Chronic pain: a review of its epidemiology and associated factors in population-based studies. *Br. J. Anaesth.* 123 e273–e283. 10.1016/j.bja.2019.03.023 31079836 PMC6676152

[B37] Mole T. B. MacIver K. Sluming V. Ridgway G. R. Nurmikko T. J. (2014). Specific brain morphometric changes in spinal cord injury with and without neuropathic pain. *Neuroimage Clin.* 5 28–35. 10.1016/j.nicl.2014.05.014 24936434 PMC4055864

[B38] Neeb L. Bastian K. Villringer K. Israel H. Reuter U. Fiebach J. B. (2017). Structural gray matter alterations in chronic migraine: implications for a progressive disease? *Headache* 57 400–416. 10.1111/head.13012 28028808

[B39] Pan P. L. Zhong J. G. Shang H. F. Zhu Y. L. Xiao P. R. Dai Z. Y. (2015). Quantitative meta-analysis of grey matter anomalies in neuropathic pain. *Eur. J. Pain* 19 1224–1231. 10.1002/ejp.670 25708697

[B40] Park J. S. Jung Y. W. (2021). Peeled images and sectioned images from real-color volume models of foot. *Surg. Radiol. Anat.* 43 37–43. 10.1007/s00276-020-02534-3 32676743

[B41] Park L. T. Zarate C. J. (2019). Depression in the primary care setting. *N. Engl. J. Med.* 380 559–568. 10.1056/NEJMcp1712493 30726688 PMC6727965

[B42] Picó-Pérez M. Moreira P. S. de Melo Ferreira V. Radua J. Mataix-Cols D. Sousa N. (2020). Modality-specific overlaps in brain structure and function in obsessive-compulsive disorder: multimodal meta-analysis of case-control MRI studies. *Neurosci. Biobehav. Rev.* 112 83–94. 10.1016/j.neubiorev.2020.01.033 32006553

[B43] Porreca F. Navratilova E. (2017). Reward, motivation, and emotion of pain and its relief. *Pain* 158 S43–S49. 10.1097/j.pain.0000000000000798 28106670 PMC5350036

[B44] Radua J. Mataix-Cols D. Phillips M. L. El-Hage W. Kronhaus D. M. Cardoner N. (2012). A new meta-analytic method for neuroimaging studies that combines reported peak coordinates and statistical parametric maps. *Eur. Psychiatry* 27 605–611. 10.1016/j.eurpsy.2011.04.001 21658917

[B45] Radua J. Rubia K. Canales-Rodríguez E. J. Pomarol-Clotet E. Fusar-Poli P. Mataix-Cols D. (2014). Anisotropic kernels for Coordinate-Based Meta-Analyses of neuroimaging studies. *Front. Psychiatry* 5:13. 10.3389/fpsyt.2014.00013 24575054 PMC3919071

[B46] Rizvi S. J. Gandhi W. Salomons T. (2021). Reward processing as a common diathesis for chronic pain and depression. *Neurosci. Biobehav. Rev.* 127 749–760. 10.1016/j.neubiorev.2021.04.033 33951413

[B47] Robinson M. E. Craggs J. G. Price D. D. Perlstein W. M. Staud R. (2011). Gray matter volumes of pain-related brain areas are decreased in fibromyalgia syndrome. *J. Pain* 12 436–443. 10.1016/j.jpain.2010.10.003 21146463 PMC3070837

[B48] Rotenstein L. S. Ramos M. A. Torre M. Segal J. B. Peluso M. J. Guille C. (2016). Prevalence of depression, depressive symptoms, and suicidal ideation among medical students. *JAMA* 316:2214. 10.1001/jama.2016.17324 27923088 PMC5613659

[B49] Schmidt-Wilcke T. Hierlmeier S. Leinisch E. (2010). Altered regional brain morphology in patients with chronic facial pain. *Headache* 50 1278–1285. 10.1111/j.1526-4610.2010.01637.x 20236343

[B50] Schmidt-Wilcke T. Leinisch E. Gänbauer S. Draganski B. Bogdahn U. Altmeppen J. (2006). Affective components and intensity of pain correlate with structural differences in gray matter in chronic back pain patients. *Pain* 125 89–97. 10.1016/j.pain.2006.05.004 16750298

[B51] Seminowicz D. A. Shpaner M. Keaser M. L. Krauthamer G. M. Mantegna J. Dumas J. A. (2013). Cognitive-Behavioral therapy increases prefrontal cortex gray matter in patients with chronic pain. *J. Pain* 14 1573–1584. 10.1016/j.jpain.2013.07.020 24135432 PMC3874446

[B52] Serrano-Sosa M. Sampathgiri K. Spuhler K. D. DeLorenzo C. Parsey R. Huang C. (2020). The importance of identifying functional Val158Met polymorphism in catechol-O-Methyltransferase when assessing MRI-based volumetric measurements in major depressive disorder. *Brain Imaging Behav.* 14 2762–2770. 10.1007/s11682-019-00225-1 31898087

[B53] Sheng J. Liu S. Wang Y. Cui R. Zhang X. (2017). The link between depression and chronic pain: neural mechanisms in the brain. *Neural Plast.* 2017 1–10. 10.1155/2017/9724371 28706741 PMC5494581

[B54] Sheng L. Ma H. Shi Y. Dai Z. Zhong J. Chen F. (2020). Cortical thickness in migraine: a Coordinate-Based Meta-Analysis. *Front. Neurosci.* 14:600423. 10.3389/fnins.2020.600423 33488349 PMC7815689

[B55] Sheng L. Zhao P. Ma H. Radua J. Yi Z. Shi Y. (2021). Cortical thickness in Parkinson’s disease: a coordinate-based meta-analysis. *Medicine* 13 4007–4023. 10.18632/aging.202368 33461168 PMC7906199

[B56] Shi H. Yuan C. Dai Z. Ma H. Sheng L. (2016). Gray matter abnormalities associated with fibromyalgia: a meta-analysis of voxel-based morphometric studies. *Semin. Arthritis Rheum.* 46 330–337. 10.1016/j.semarthrit.2016.06.002 27989500

[B57] Smallwood R. F. Laird A. R. Ramage A. E. Parkinson A. L. Lewis J. Clauw D. J. (2013). Structural brain anomalies and chronic pain: a quantitative meta-analysis of gray matter volume. *J. Pain* 14 663–675. 10.1016/j.jpain.2013.03.001 23685185 PMC4827858

[B58] Tang Y. Wang M. Zheng T. Yuan F. Yang H. Han F. (2020). Grey matter volume alterations in trigeminal neuralgia: a systematic review and meta-analysis of voxel-based morphometry studies. *Prog. Neuropsychopharmacol. Biol. Psychiatry* 98:109821. 10.1016/j.pnpbp.2019.109821 31756417

[B59] Wang Y. Cao D. Y. Remeniuk B. Krimmel S. Seminowicz D. A. Zhang M. (2017). Altered brain structure and function associated with sensory and affective components of classic trigeminal neuralgia. *Pain* 158 1561–1570. 10.1097/j.pain.0000000000000951 28520647

[B60] Wise T. Radua J. Via E. Cardoner N. Abe O. Adams T. (2017). Common and distinct patterns of grey-matter volume alteration in major depression and bipolar disorder: evidence from voxel-based meta-analysis. *Mol. Psychiatry* 22 1455–1463. 10.1038/mp.2016.72 27217146 PMC5622121

[B61] Yalcin I. Barrot M. (2014). The anxiodepressive comorbidity in chronic pain. *Curr. Opin. Anaesthesiol.* 27 520–527. 10.1097/ACO.0000000000000116 25188218

[B62] Zhang Y. Mao Z. Pan L. Ling Z. Liu X. Zhang J. (2018). Dysregulation of pain- and Emotion-Related networks in trigeminal neuralgia. *Front. Hum. Neurosci.* 12:107. 10.3389/fnhum.2018.00107 29662445 PMC5890150

