# Supplementary material for: Gray Matter Volume Abnormality in Chronic Pain Patients With Depressive Symptoms: A Systemic Review and Meta-Analysis of Voxel-Based Morphometry Studies
Source: Front Neurosci. 2022 Jun 6;16:826759. doi: 10.3389/fnins.2022.826759 (PMC9207409; doi:10.3389/fnins.2022.826759)
Supplement: Supplementary file 1 [file Table_1.docx]

Supplementary Material

**Contents**

Data S1. Systematic search strategy………….…………………………………….2

Table S1. Checklists of PRISMA statement for reporting systematic reviews and meta-analyses……………………………………………………………………….3

Table S2. Quality assessment by 12-point score of the 18 studies included…..…....5

Table S3. The detail depressive symptom information of 18 included studies….….7

Table S4. Heterogeneity assessment of main analysis results of total 18 studies included……………………………………………………………………………..8

Table S5. Sensitivity assessment by Jackknife analysis of main results in total 18 studies included…………………………………………...…………………..…….9

Table S6. Sensitivity assessment by Jackknife analysis of subgroup results………10

**Data S1. Systematic search strategy (Deadline on July 13, 2021)**

**PubMed (30):** ((chronic pain [Text Word]) AND ((depressive [Text Word]) OR (depression [Text Word]))) AND ((((VBM [Text Word]) OR (voxel-based morphometry [Text Word])) OR (gray matter [Text Word])) OR (gray matter volume [Text Word]))

**MEDLINE (1542):** (chronic pain).tw. AND (depressive or depression).tw. AND (VBM or voxel-based morphometry or gray matter volume or gray matter).tw.

**Web of Science (143):** (TS=chronic pain) AND (TS=depressive OR TS=depression) AND (TS=VBM OR TS= voxel-based morphometry OR TS= gray matter volume OR TS= gray matter)

**Cochrane library (7):** ((chronic pain): ti,ab,kw) AND ((depressive):ti,ab,kw OR (depression):ti,ab,kw) AND ((VBM):ti,ab,kw OR (voxel-based morphometry):ti,ab,kw OR (gray matter volume):ti,ab,kw OR (gray matter):ti,ab,kw)

**Table S1. Checklists of PRISMA statement for reporting systematic reviews and meta-analyses**

| **Section/topic** | **Item No** | **Checklist item** | **Reported on page No** |  |
| --- | --- | --- | --- | --- |
| **Title** | | | | |
| Title | 1 | Identify the report as a systematic review, meta-analysis, or both | 1 |  |
| **Abstract** | | | | |
| Structured summary | 2 | Provide a structured summary including, as applicable, background, objectives, data  sources, study eligibility criteria, participants, interventions, study appraisal and synthesis  methods, results, limitations, conclusions and implications of key findings, systematic  review registration number | 1-2 |  |
| **Introduction** | | | |  |
| Rationale | 3 | Describe the rationale for the review in the context of what is already known | 2 |  |
| Objectives | 4 | Provide an explicit statement of questions being addressed with reference to participants,  interventions, comparisons, outcomes, and study design (PICOS) | 2 |  |
| **Methods** | | | |  |
| Protocol and registration | 5 | Indicate if a review protocol exists, if and where it can be accessed (such as web address),  and, if available, provide registration information including registration number | 2 |  |
| Eligibility criteria | 6 | Specify study characteristics (such as PICOS, length of follow-up) and report characteristics (such as years considered, language, publication status) used as criteria for eligibility, giving rationale | 2 |  |
| Information sources | 7 | Describe all information sources (such as databases with dates of coverage, contact with  study authors to identify additional studies) in the search and date last searched | 2 |  |
| Search | 8 | Present full electronic search strategy for at least one database, including any limits used,  such that it could be repeated | 2 |  |
| Study selection | 9 | State the process for selecting studies (that is, screening, eligibility, included in systematic  review, and, if applicable, included in the meta-analysis) | 2-3 |  |
| Data collection process | 10 | Describe method of data extraction from reports (such as piloted forms, independently, in  duplicate) and any processes for obtaining and confirming data from investigators | 2-3 |  |
| Data items | 11 | List and define all variables for which data were sought (such as PICOS, funding sources) and any assumptions and simplifications made | 3 |  |
| Risk of bias in individual studies | 12 | Describe methods used for assessing risk of bias of individual studies (including  specification of whether this was done at the study or outcome level), and how this  information is to be used in any data synthesis | 3-6 |  |
| Summary measures | 13 | State the principal summary measures (such as risk ratio, difference in means). | 3 |  |
| Synthesis of results | 14 | Describe the methods of handling data and combining results of studies, if done, including  measures of consistency (such as I2) for each meta-analysis | 3 |  |
| Risk of bias across studies | 15 | Specify any assessment of risk of bias that may affect the cumulative evidence (such as  publication bias, selective reporting within studies) | 3-6 |  |
| Additional analyses | 16 | Describe methods of additional analyses (such as sensitivity or subgroup analyses, meta-regression), if done, indicating which were pre-specified | 6 |  |
| **Results** | | | |  |
| Study selection | 17 | Give numbers of studies screened, assessed for eligibility, and included in the review, with reasons for exclusions at each stage, ideally with a flow diagram | 6 |  |
| Study characteristics | 18 | For each study, present characteristics for which data were extracted (such as study size,  PICOS, follow-up period) and provide the citations | 6-7 |  |
| Risk of bias within studies | 19 | Present data on risk of bias of each study and, if available, any outcome-level assessment  (see item 12). | 7 |  |
| Results of individual studies | 20 | For all outcomes considered (benefits or harms), present for each study (a) simple summary data for each intervention group and (b) effect estimates and confidence intervals, ideally with a forest plot | 7 |  |
| Synthesis of results | 21 | Present results of each meta-analysis done, including confidence intervals and measures of consistency | 7 |  |
| Risk of bias across studies | 22 | Present results of any assessment of risk of bias across studies (see item 15) | 7 |  |
| Additional analysis | 23 | Give results of additional analyses, if done (such as sensitivity or subgroup analyses, meta-regression [see item 16]) | 7 |  |
| Summary of evidence | 24 | Summarise the main findings including the strength of evidence for each main outcome;  consider their relevance to key groups (such as health care providers, users, and policy  makers) | 7-9 |  |
| Limitations | 25 | Discuss limitations at study and outcome level (such as risk of bias), and at review level (such as incomplete retrieval of identified research, reporting bias) | 9 |  |
| Conclusions | 26 | Provide a general interpretation of the results in the context of other evidence, and  implications for future research | 9 |  |
| **Funding** | | | |  |
| Funding | 27 | Describe sources of funding for the systematic review and other support (such as supply of data) and role of funders for the systematic review | 9 |  |

**Table S2.** **Quality assessment by 12-point score of the 18 studies included**

| **Part 1. Subjects** | **Part 2. Methods** | **Part 3. Results and conclusions** |
| --- | --- | --- |
| 1. Patients were evaluated prospectively, specific diagnostic criteria were applied, and demographic data were reported (0 or 1) 2. Healthy comparison participants were evaluated prospectively; psychiatric and medical illnesses were excluded (0 or 1) 3. Important variables (e.g., age, gender, disease duration, depressive symptom score, and types of chronic) were checked either via stratification or statistics (0.5 or 1) 4. State all patients were no intervention during the study (0 or 1) 5. All patients performed depressive symptom assessment (0 or 1) 6. Sample size per group: ≥ 10; ≥ 20 (0.5 or 1) | 1. Magnet strength: 1.5 T; 3.0T (0.5 or 1) 2. The imaging technique used was clearly described so that it could be reproduced (0 or 1) 3. Whole brain analysis was automated without a previously defined region (0 or 1) 4. Spatial coordinates were reported in a standard space (e.g., Talairach or MNI coordinates) (0.5 or 1) | 1. Statistical results were corrected for multiple comparison (0.5 or 1) 2. Conclusions were consistent with the results obtained, and the limitations were discussed (0.5 or 1) |
| **Total score of the three parts: > 6.0 will be included.** | | |

| **Studies** | **Part 1. Subject** | | | | | | **Part 2. Methods** | | | | **Part 3. Results and conclusions** | | **Total**  **score** |
| --- | --- | --- | --- | --- | --- | --- | --- | --- | --- | --- | --- | --- | --- |
|  | **⑴** | **⑵** | **⑶** | **⑷** | **⑸** | **⑹** | **⑺** | **⑻** | **⑼** | **⑽** | **⑾** | **⑿** |  |
| David et al | 1 | 1 | 0.5 | 1 | 1 | 0.5 | 1 | 1 | 1 | 1 | 1 | 1 | 11 |
| Fayed et al | 1 | 1 | 0.5 | 1 | 1 | 0.5 | 0.5 | 1 | 1 | 1 | 1 | 1 | 10.5 |
| Ikeda et al | 1 | 1 | 1 | 1 | 1 | 0.5 | 1 | 1 | 1 | 1 | 0.5 | 1 | 11 |
| James et al | 1 | 1 | 1 | 0 | 1 | 1 | 1 | 1 | 1 | 1 | 1 | 1 | 11 |
| Liao et al | 1 | 1 | 1 | 0 | 1 | 1 | 1 | 1 | 1 | 1 | 1 | 1 | 11 |
| Mao et al | 1 | 1 | 1 | 0 | 1 | 1 | 1 | 1 | 0 | 1 | 1 | 1 | 10 |
| Markus et al | 1 | 1 | 0.5 | 0 | 1 | 0.5 | 1 | 1 | 0 | 1 | 0.5 | 1 | 8.5 |
| Michael et al | 1 | 1 | 0.5 | 0 | 1 | 0.5 | 1 | 1 | 0 | 1 | 1 | 1 | 9 |
| Mole et al | 1 | 1 | 1 | 0 | 1 | 0.5 | 1 | 1 | 1 | 1 | 0.5 | 1 | 10 |
| Sawsan et al | 1 | 1 | 0.5 | 1 | 1 | 0.5 | 1 | 1 | 0 | 1 | 1 | 1 | 10 |
| Shariq et al | 1 | 1 | 1 | 1 | 1 | 0.5 | 1 | 1 | 1 | 1 | 1 | 1 | 11.5 |
| Tobias_1 et al | 1 | 1 | 1 | 0 | 1 | 0.5 | 0.5 | 1 | 1 | 0.5 | 1 | 1 | 9.5 |
| Wang et al | 1 | 1 | 1 | 0 | 1 | 1 | 1 | 1 | 1 | 1 | 1 | 0.5 | 10.5 |
| Tobias_2 et al | 1 | 1 | 1 | 0 | 1 | 0.5 | 0.5 | 1 | 1 | 1 | 0.5 | 0.5 | 9 |
| Zhang et al | 1 | 1 | 1 | 1 | 1 | 1 | 1 | 1 | 1 | 1 | 1 | 1 | 12 |
| Fritz et al | 1 | 1 | 0.5 | 0 | 1 | 1 | 0.5 | 1 | 1 | 1 | 1 | 1 | 10 |
| Hubbard et al | 1 | 1 | 0.5 | 0 | 1 | 0.5 | 1 | 1 | 1 | 1 | 1 | 1 | 10 |
| Neeb et al | 1 | 1 | 1 | 1 | 1 | 1 | 1 | 1 | 1 | 1 | 1 | 1 | 12 |

**Table S3. The detail depressive symptom information of 18 included studies**

| **Studies** | **Tool of depressive symptom assessment** | **Score (mean ± sd.)** | | **P value** |
| --- | --- | --- | --- | --- |
|  |  | **PT** | **HC** |  |
| **David et al** | BDI | 19.85 (2.79) | NA | NA |
| **Fayed et al** | HADS | 5.90 (3.80) | 1.60 (0.51) | 0.001 |
| **Ikeda et al** | BDI | 16.50 (12.4) | 3.20 (4.70) | <0.001 |
| **James et al** | BDI | 15.36 (8.28) | 3.32 (4.73) | NA |
| **Mao et al** | HAMD (LBP) | 8.00 (5.00) | 2.90 (2.50) | 0.000 |
|  | HAMD (UBP) | 9.60 (5.60) | 3.10 (2.60) | 0.000 |
| **Markus et al** | HADS | 16.90 (7.40) | 8.40 (4.40) | 0.001 |
| **Michael et al** | BDI | 34.38 (23.86) | 4.00 (9.66) | 0.001 |
| **Mole et al** | BDI | 12.39 (NA) | NA | NA |
| **Sawsan et al** | CES-D  (With endometriosis) | 12.50 (7.20) | 2.40 (2.80) | <0.001 |
|  | CES-D  (Without endometriosis) | 8.80 (2.60) | 1.2 (2.1) | <0.001 |
| **Shariq et al** | BDI | 9.10 (4.90) | 1.80 (2.50) | 0.001 |
| **Tobias_1 et al** | BDI★ | 11.38 (2.05) ^*^ | NA | NA |
| **Wang et al** | HAMD | 4.24 (3.37) | 0.34 (0.85) | 0.000 |
| **Tobias_2 et al** | HAMD☆ | 10.00 (0.98) ^*^ | NA | NA |
| **Zhang et al** | HAMD | 3.79 (1.76) | 0.29 (0.46) | <0.001 |
| **Fritz et al** | PHQ9 | 4.49 (2.68) | 2.42 (2.29) | <0.05 |
| **Hubbard et al** | POMS | 17.35 (2.50) | 15.61 (1.14) | 0.011 |
| **Neeb et al** | BDI | 13.0 (10.1) | 2.9 (3.0) | <0.001 |

*: Calculated by SPSS 19.0 according to the material of the studies; NA: Not available.

★: According to the criterion by the study statement (Schmidt-Wilcke et al., 2010)

☆: According to the standard criterion (Carrozzino et al., 2020)

| **Studies** | **Tool of depressive symptom assessment** | **Score (median)** | | **P value** |
| --- | --- | --- | --- | --- |
|  |  | **PT** | **HC** |  |
| **Liao et al** | HAMD | 6.00 (4.00,7.00) | 3.00 (1.80,4.00) | <0.001 |

**Table S4. Heterogeneity assessment of main analysis results of the 18 studies included**

1. **Template: gray_matter. Anisotropy: 1.0. Isotropic FWHM: 20mm. Mask: gray_matter**

| **Study** | **Mask** | **Maximum** | |  | **Minimum** | |
| --- | --- | --- | --- | --- | --- | --- |
|  |  | **MNI** | **Region** |  | **MNI** | **Region** |
| David | Whole volume | (none) | |  | (none) | |
|  |  | (none) | |  | (none) | |
| Fayed | Whole volume | 24, -18, -18 | Right parahippocampal gyrus, BA 20 |  | (none) | |
|  |  | 24, -18, -18 | Right parahippocampal gyrus, BA 20 |  | (none) | |
| Michael | Whole volume | (none) | |  | -2, -10,44 | Left median cingulate / paracingulate gyri, BA 23 |
|  |  | (none) | |  | -2, -10,44 | Left median cingulate / paracingulate gyri, BA 23 |
| Ikeda | Whole volume | (none) | |  | 32,10, -18 | Right insula |
|  |  | (none) | |  | 32,10, -18 | Right insula |
| James | Whole volume | 46,60,28 | (undefined) |  | 32,20,28 | Right superior longitudinal fasciculus II |
|  |  | 36,58,22 | Right middle frontal gyrus |  | 32,20,28 | Right superior longitudinal fasciculus II |
| Liao | Whole volume | (none) | |  | (none) | |
|  |  | (none) | |  | (none) | |
| Mao_1 | Whole volume | 12,6, -10 | (undefined), BA 25 |  | 8, -74,24 | Corpus callosum |
|  |  | 12,6, -10 | (undefined), BA 25 |  | 8, -74,24 | Corpus callosum |
| Markus | Whole volume | (none) | |  | -26,4, -20 | Left temporal pole, superior temporal gyrus, BA 34 |
|  |  | (none) | |  | -26,4, -20 | Left temporal pole, superior temporal gyrus, BA 34 |
| Mole | Whole volume | (none) | |  | (none) | |
|  |  | (none) | |  | (none) | |
| Shariq | Whole volume | -34, -36, -10 | Left inferior network, inferior longitudinal fasciculus |  | (none) | |
|  |  | -34, -36, -10 | Left inferior network, inferior longitudinal fasciculus |  | (none) | |
| Tobias_1 | Whole volume | (none) | |  | (none) | |
|  |  | (none) | |  | (none) | |
| Wang | Whole volume | 28, -74,48 | Right superior parietal gyrus, BA 7 |  | -50, -48, -22 | Left inferior temporal gyrus, BA 20 |
|  |  | 28, -74,48 | Right superior parietal gyrus, BA 7 |  | -50, -48, -22 | Left inferior temporal gyrus, BA 20 |
| Tobias_2 | Whole volume | 32, -16, -2 | Corpus callosum |  | 52, -28, -30 | (undefined) |
|  |  | 32, -16, -2 | Corpus callosum |  | 52, -28, -30 | (undefined) |
| Zhang | Whole volume | (none) | |  | 18,4, -22 | Right parahippocampal gyrus, BA 34 |
|  |  | (none) | |  | 18,4, -22 | Right parahippocampal gyrus, BA 34 |
| Sawsan_2 | Whole volume | (none) | |  | -12, -20,12 | Left anterior thalamic projections |
|  |  | (none) | |  | -12, -20,12 | Left anterior thalamic projections |
| Sawsan_1 | Whole volume | -20, -2, -24 | Left amygdala, BA 28 |  | 44, -36,16 | Right superior longitudinal fasciculus III |
|  |  | -20, -2, -24 | Left amygdala, BA 28 |  | 44, -36,16 | Right superior longitudinal fasciculus III |
| Mao_2 | Whole volume | (none) | |  | -34, -20,66 | Left precentral gyrus, BA 6 |
|  |  | (none) | |  | -34, -20,66 | Left precentral gyrus, BA 6 |
| Fritz | Whole volume | (none) | |  | 16,48, -12 | Right striatum |
|  |  | (none) | |  | 16,50, -12 | Right striatum |
| Hubbard | Whole volume | -22, -16, -16 | Left hippocampus |  | -42, -28,54 | Left postcentral gyrus, BA 4 |
|  |  | -22, -16, -16 | Left hippocampus |  | -42, -28,54 | Left postcentral gyrus, BA 4 |
| Neeb | Whole volume | 34, -4, -10 | (undefined), BA 48 |  | (none) | |
|  |  | 34, -4, -10 | (undefined), BA 48 |  | (none) | |

1. **Assessment of residual heterogeneity**
2. **Positive peaks (τ = 0.244, Q = 24.798, df = 8, P < 0.005)**

| **Study** | ***d*** | ***SE*** | ***z*** | ***P*** | ***CI_low_*** | ***CI_up_*** |
| --- | --- | --- | --- | --- | --- | --- |
|  |  |  |  |  |  |  |
| Fayed | 2.647 | 0.597 | 4.436 | 0.000009179 | 1.477 | 3.817 |
| James | 0.419 | 0.216 | 1.942 | 0.052104831 | -0.004 | 0.843 |
| Mao_1 | 0.669 | 0.266 | 2.516 | 0.011859775 | 0.148 | 1.190 |
| Shariq | 1.957 | 0.597 | 3.278 | 0.001045704 | 0.787 | 3.127 |
| Wang | 0.749 | 0.238 | 3.152 | 0.001621366 | 0.283 | 1.215 |
| Tobias_2 | 1.317 | 0.371 | 3.545 | 0.000393152 | 0.589 | 2.045 |
| Sawsan_1 | 1.537 | 0.396 | 3.883 | 0.000103116 | 0.761 | 2.313 |
| Hubbard | 1.398 | 0.382 | 3.663 | 0.000249505 | 0.650 | 2.146 |
| Neeb | 1.371 | 0.346 | 3.961 | 0.000074744 | 0.692 | 2.049 |
| Mean | 1.197 | 0.205 | 5.825 | 0.000000000 | 0.794 | 1.599 |

1. **Negative peaks (τ = 0.101, Q = 30.891, df = 12, P < 0.005)**

| **Study** | ***d*** | ***SE*** | ***z*** | ***P*** | ***CI_low_*** | ***CI_up_*** |
| --- | --- | --- | --- | --- | --- | --- |
| Michael | -0.959 | 0.429 | -2.237 | 0.025314201 | -1.800 | -0.119 |
| Ikeda | -1.260 | 0.352 | -3.576 | 0.000349135 | -1.951 | -0.569 |
| James | -0.474 | 0.217 | -2.187 | 0.028708842 | -0.898 | -0.049 |
| Mao_1 | -0.538 | 0.263 | -2.045 | 0.040810633 | -1.054 | -0.022 |
| Markus | -1.040 | 0.406 | -2.558 | 0.010515321 | -1.836 | -0.243 |
| Wang | -0.852 | 0.240 | -3.550 | 0.000385314 | -1.323 | -0.382 |
| Tobias_2 | -1.417 | 0.377 | -3.757 | 0.000171705 | -2.156 | -0.678 |
| Zhang | -0.994 | 0.269 | -3.700 | 0.000216017 | -1.521 | -0.467 |
| Sawsan_2 | -2.733 | 0.715 | -3.820 | 0.000133422 | -4.135 | -1.331 |
| Sawsan_1 | -1.372 | 0.386 | -3.558 | 0.000373294 | -2.128 | -0.616 |
| Mao_2 | -0.865 | 0.384 | -2.252 | 0.024334202 | -1.618 | -0.112 |
| Fritz | -0.453 | 0.107 | -4.222 | 0.000024234 | -0.663 | -0.243 |
| Hubbard | -1.490 | 0.387 | -3.849 | 0.000118530 | -2.249 | -0.731 |
| Mean | -0.945 | 0.126 | -7.503 | 0.000000000 | -1.191 | -0.698 |

**Table S5. Sensitivity assessment by Jackknife analysis (main results in total 18 studies included).**

1. **Gray matter volume (GMV): Chronic pain patients with depressive symptom > Healthy controls (HCs)**

| **Brain area** | David  et al | Fayed  et al | Fritz  et al | Hubbard  et al | Ikeda  et al | James  et al | Liao  et al | Mao_1  et al | Mao_2  et al | Markus  et al | Michael  et al | Mole  et al | Neeb  et al | Sawsan_1  et al | Sawsan_2  et al | Shariq  et al | Tobias_1  et al | Wang  et al | Tobias_2  et al | Zhang  et al | Total |
| --- | --- | --- | --- | --- | --- | --- | --- | --- | --- | --- | --- | --- | --- | --- | --- | --- | --- | --- | --- | --- | --- |
| Left hippocampus | Y | N | Y | N | Y | Y | Y | Y | Y | Y | Y | Y | Y | Y | Y | N | Y | Y | N | Y | 16/20 |

1. **Gray matter volume (GMV): Healthy controls (HCs) > Chronic pain patients with depressive symptom**

| **Brain area** | David  et al | Fayed  et al | Fritz  et al | Hubbard  et al | Ikeda  et al | James  et al | Liao  et al | Mao_1  et al | Mao_2  et al | Markus  et al | Michael  et al | Mole  et al | Neeb  et al | Sawsan_1  et al | Sawsan_2  et al | Shariq  et al | Tobias_1  et al | Wang  et al | Tobias_2  et al | Zhang  et al | Total |
| --- | --- | --- | --- | --- | --- | --- | --- | --- | --- | --- | --- | --- | --- | --- | --- | --- | --- | --- | --- | --- | --- |
| Left superior frontal gyrus, medial, BA 10 | Y | Y | N | Y | Y | Y | Y | Y | Y | Y | Y | Y | Y | Y | Y | Y | Y | Y | Y | Y | 19/20 |
| Left inferior network, uncinate fasciculus | Y | Y | N | Y | Y | Y | Y | Y | Y | Y | Y | Y | Y | Y | Y | Y | Y | Y | Y | Y | 19/20 |

**Table S6. Jackknife analysis of subgroup results**

1. **Gray matter abnormality in Neuropathic pain**
2. **chronic pain with depressive symptom > healthy controls**

| **Brain area** | Fritz  et al | Hubbard  et al | Mao_1  et al | Mao_2  et al | Mole  et al | Neeb  et al | Shariq  et al | Tobias_1  et al | Wang  et al | Tobias_2  et al | Zhang  et al | Total |
| --- | --- | --- | --- | --- | --- | --- | --- | --- | --- | --- | --- | --- |
| Right inferior network, inferior fronto-occipital fasciculus | Y | Y | Y | Y | Y | N | Y | Y | Y | N | N | 8/11 |
| Left lenticular nucleus, putamen, BA 34 | N | N | N | N | N | N | N | N | N | N | N | 0/11 |

1. **healthy controls > chronic pain with depressive symptom**

| **Brain area** | Fritz  et al | Hubbard  et al | Mao_1  et al | Mao_2  et al | Mole  et al | Neeb  et al | Shariq  et al | Tobias  et al | Wang  et al | Tobias_2  et al | Zhang  et al | Total |
| --- | --- | --- | --- | --- | --- | --- | --- | --- | --- | --- | --- | --- |
| Right anterior cingulate / paracingulate gyri, BA 10 | N | N | N | N | N | N | N | N | N | N | N | 0/11 |
| Right superior frontal gyrus, medial orbital, BA 10 | N | Y | Y | Y | Y | Y | N | Y | N | Y | Y | 8/11 |
| Left inferior network, uncinate fasciculus | N | Y | Y | Y | Y | Y | Y | Y | Y | Y | Y | 10/11 |

1. **Gray matter abnormality in Musculoskeletal pain**
   1. **chronic pain with depressive symptom > healthy controls**

| **Brain area** | Fayed  et al | James  et al | Markus  et al | Michael  et al | Total |
| --- | --- | --- | --- | --- | --- |
| Right parahippocampal gyrus, BA 35 | N | Y | Y | Y | 3/4 |
| Left hippocampus, BA 20 | N | Y | Y | Y | 3/4 |
| Right middle frontal gyrus | Y | N | Y | Y | 3/4 |

| **Brain area** | Fayed  et al | James  et al | Markus  et al | Michael  et al | Total |
| --- | --- | --- | --- | --- | --- |
| (undefined), BA 34 | Y | Y | N | N | 2/4 |
| Right superior longitudinal fasciculus II | Y | N | Y | Y | 3/4 |

- 1. **healthy controls > chronic pain with depressive symptom**

**References:**

Carrozzino, D., Patierno, C., Fava, G.A., and Guidi, J. (2020). The hamilton rating scales for depression: A critical review of clinimetric properties of different versions. *Psychother Psychosom.* 89, 133-150. doi:10.1159/000506879

Schmidt-Wilcke, T., Hierlmeier, S., and Leinisch, E. (2010). Altered regional brain morphology in patients with chronic facial pain. *Headache: The Journal of Head and Face Pain* 50, 1278-1285. doi:10.1111/j.1526-4610.2010. 01637.x
