# Supplementary material for: Gray Matter Volume Abnormality in Chronic Pain Patients With Depressive Symptoms: A Systemic Review and Meta-Analysis of Voxel-Based Morphometry Studies
Source: Front Neurosci. 2022 Jun 6;16:826759. doi: 10.3389/fnins.2022.826759 (PMC9207409; doi:10.3389/fnins.2022.826759)
Supplement: Supplementary file 2 [file Table_2.DOCX]

| **Coordinate information and their original studies** | | |
| --- | --- | --- |
| **Number** | **File name** | **Coordinate information (x, y, z, t value)** |
| **1** | **David.no_peaks.txt** | **None** |
| *Seminowicz, D. A., et al. (2013) Cognitive-Behavioral Therapy Increases Prefrontal Cortex Gray Matter in Patients With Chronic Pain, The Journal of Pain, 14, 1573-1584.* | | |
| **2** | **Fayed.spm_mni.txt** | **23, -17, -17, 6.85**  **-27, -20, -15, 6.31** |
| *Fayed, N., et al. (2017) Difference in Regional Brain Volume between Fibromyalgia Patients and Long-Term Meditators, Actas Esp Psiquiatr, 45, 268-76.* | | |
| **3** | **Ikeda.spm_mni.txt** | **32, 10, -18, -4.23**  **-4, -15, 38, -3.81** |
| *Ikeda, E., et al. (2018) Anterior insular volume decrease is associated with dysfunction of the reward system in patients with chronic pain, European journal of pain, 22, 1170-1179.* | | |
| **4** | **James.fsl_mni.txt** | **44, 59, 30, 2.50**  **37, 59, 27, 2.00**  **38, 73, 42, 1.73**  **42, 66, 33, 1.67**  **32, 19, 30, -2.23** |
| *James H., et al. (2018) Structural network differences in chronic muskuloskeletal pain: Beyond fractional anisotropy, NeuroImage.* | | |
| **5** | **Liao.no_peaks.txt** | **None** |
| *Liao, X., et al. (2018) Brain gray matter alterations in Chinese patients with chronic knee osteoarthritis pain based on voxel-based morphometry, Medicine, 97, e0145.* | | |
| **6** | **Mao_1.fsl_mni.txt** | **-30, 4, 2, 2.32**  **16, 6, -6, 2.85**  **16, 6, -4, 2.66**  **-10, 6, -8, 2.85**  **12, 6, -8, 3.24**  **-14, 6, -14, 2.25**  **8, 8, -2, 2.20**  **-24, -38, 42, -1.85**  **-18, -42, 44, -1.75**  **-22, -70, 22, -2.04**  **8, -74, 24, -3.00**  **-16, -42, 40, -1.92** |
|  | **Mao_2.fsl_mni.txt** | **-34, -22, 66, -2.75**  **-34, -24, 66, -2.12** |
| *Mao, C., et al. (2013) Differences in brain structure in patients with distinct sites of chronic pain: A voxel-based morphometric analysis, Neural Regen Res, 8, 2981-90.* | | |
| **7** | **Markus.spm_mni.txt** | **14, 46, 6, -3.20**  **57, 38, 4, -2.10**  **-27, 4, -20, -3.20** |
| *Burgmer, M., et al. (2009) Decreased Gray Matter Volumes in the Cingulo-Frontal Cortex and the Amygdala in Patients With Fibromyalgia, Psychosomatic Medicine, 71, 566-573.* | | |
| **8** | **Michael.spm_tal.txt** | **-34, -2, 8, -2.244**  **-3, 11, 31, -2.347**  **-3, -12, 42, -2.796** |
| *Robinson, M., et al. (2011) Gray matter volumes of pain-related brain areas are decreased in fibromyalgia syndrome, J Pain, 12, 436-43.* | | |
| **9** | **Mole.no_peaks.txt** | **None** |
| *Mole, T. B., et al. (2014) Specific brain morphometric changes in spinal cord injury with and without neuropathic pain, NeuroImage: Clinical, 5, 28-35.* | | |
| **10** | **Sawsan_1.spm_mni.txt** | **-13, -16, 7, -4.49**  **-37,34, 26, -4.26**  **-2,11, 30, -3.73**  **4, 2, 40, -3.83**  **29, 0, -3, -3.63**  **45, -35, 17, -4.65**  **-21, -1, -23,4.59** |
|  | **Sawsan_2.spm_mni.txt** | **-13, -20, 12, -5.74** |
| *As-Sanie, S., et al. (2012) Changes in regional gray matter volume in women with chronic pelvic pain: A voxel-based morphometry study, Pain, 153, 1006-1014.* | | |
| **11** | **Shariq.spm_mni.txt** | **-34, -36, -9, 4.47**  **-8, 56, 4, 4.25** |
| *Khan, S. A., et al. (2014) Altered structure and function in the hippocampus and medial prefrontal cortex in patients with burning mouth syndrome, Pain, 155, 1472-1480.* | | |
| **12** | **Tobias.no_peaks.txt**  **(Tobias_1 et al)** | **-33, 18, -5, -5.20** |
| *Schmidt-Wilcke, T. et al. (2010) Altered Regional Brain Morphology in Patients With Chronic Facial Pain, Headache: The Journal of Head and Face Pain, 50, 1278-1285.* | | |
| **13** | **Wang.spm_mni.txt** | **-1, 38, 1, -3.85**  **-6, -3, 41, -3.52**  **-51, -48, -21, -4.68**  **53, -36, -27, -3.25**  **-54, -14, 4, -3.47**  **28, -72, 50, 3.30** |
| *Wang, Y., et al. (2017) Altered brain structure and function associated with sensory and affective components of classic trigeminal neuralgia, Pain, 158, 1561-1570.* | | |
| **14** | **Wilcke.spm_tal.txt**  **(Tobias_2 et al)** | **28, -17, 2, 4.44**  **-24, -14, 5, 4.22**  **-22, -25, 3, 3.46**  **2, -25, -18, -3.88**  **12, -38, 80, -4.26**  **38, 32, 39, -4.15**  **47, -25, -24, -5.14**  **-34, -61, -10, -4.31** |
| *Schmidt-Wilcke, T., et al. (2006) Affective components and intensity of pain correlate with structural differences in gray matter in chronic back pain patients, Pain, 125, 89-97.* | | |
| **15** | **Zhang.spm_mni.txt** | **36, 0, 18, -3.46**  **-19, 3, -21, -4.04**  **18, 4.5, -22, -4.68**  **1.5, -24, -10, -3.75** |
| *Zhang, Y., et al. (2018) Dysregulation of Pain- and Emotion-Related Networks in Trigeminal Neuralgia, Front Hum Neurosci, 12, 107.* | | |
| **16** | **Fritz.spm_mni.txt** | **15, 50, -12, -4.39**  **-27, 30, -6, -4.22**  **-6, 62, 9, -4.17** |
| *Fritz, H., et al. (2016) Chronic Back Pain Is Associated With Decreased Prefrontal and Anterior Insular Gray Matter: Results From a Population-Based Cohort Study, The Journal of Pain, 17, 111-118.* | | |
| **17** | **Hubbard.spm_mni.txt** | **-22, -15, -15, 4.23**  **-42, -27, 54, -4.62**  **-8, 56, 16, -4.39**  **-3, 30, 16, -4.52** |
| *Hubbard, C. S., et al. (2014) Altered Brain Structure and Function Correlate with Disease Severity and Pain Catastrophizing in Migraine Patients, eNeuro, 1, e20.14.* | | |
| **18** | **Neeb.spm_mni.txt** | **33, -3, -12, 6.34**  **32, 3, -32, 4.21**  **-33, -4, -11, 4.00**  **28, -49, 58, 3.56** |
| *Neeb, L., et al. (2017) Structural Gray Matter Alterations in Chronic Migraine: Implications for a Progressive Disease? Headache: The Journal of Head and Face Pain, 57, 400-416.* | | |
